# YKL-40 Is Associated With Ultrasound-Determined Carotid Atherosclerotic Plaque Instability

**DOI:** 10.3389/fneur.2021.622869

**Published:** 2021-02-17

**Authors:** Yu Wang, Bohong Li, Yong Jiang, Runhua Zhang, Xia Meng, Xingquan Zhao, Yongjun Wang, Xihai Zhao, Gaifen Liu

**Affiliations:** ^1^Department of Neurology, Beijing Tiantan Hospital, Capital Medical University, Beijing, China; ^2^Department of Rehabilitation Medicine, The People's Hospital of Xiangzhou District, Zhuhai, China; ^3^China National Clinical Research Center for Neurological Diseases, Beijing, China; ^4^Center of Stroke, Beijing Institute for Brain Disorders, Beijing, China; ^5^Beijing Key Laboratory of Translational Medicine for Cerebrovascular Disease, Beijing, China; ^6^Department of Biomedical Engineering, Center for Biomedical Imaging Research, School for Medicine, Tsinghua University, Beijing, China

**Keywords:** YKL-40, plaque instability, association, carotid plaque, atheroscelorsis

## Abstract

**Background and Aims:** YKL-40, an inflammatory biomarker, has been reported to be involved in the process and progression of atherosclerosis. Several studies have investigated the association between YKL-40 and plaque and suggested YKL-40 might be a potential biomarker for plaque instability. This study aimed to investigate the association between YKL-40 and carotid plaque instability.

**Methods:** Based on a community-based study in Beijing from February 2014 to May 2016, 1,132 participants with carotid plaques were enrolled in this study. Data on demographics and medical history were collected through face-to-face interviews, and fasting blood samples were collected and stored. We used ultrasound to evaluate the presence of carotid plaque and its instability. The level of YKL-40 was measured by enzyme-linked immunosorbent assay (ELISA). Multivariate logistic regression analysis was performed to investigate the association between YKL-40 level and carotid atherosclerotic plaque instability.

**Results:** The mean age of the 1,132 participants was 58.0 (52.0–64.0) years, and 560 (49.5%) were male. Unstable plaques were detected in 855 (75.53%) participants. YKL-40 level was classified into four groups according to its quartile: quartile 1: <25.47 ng/mL, quartile 2: 25.47–39.53 ng/mL, quartile 3: 39.53–70.55 ng/mL, quartile 4: ≥70.55 ng/mL. After adjusting for age, sex, smoking, alcohol drinking, medical history, triglycerides, low-density lipoprotein cholesterol, high-density lipoprotein cholesterol, homocysteine, high-sensitivity C-reactive protein, and plaque thickness, the top quartiles of YKL-40 level were significantly associated with unstable plaque (quartile 3: OR 2.10, 95% CI 1.29–3.40; quartile 4: OR 1.70, 95% CI 1.04–2.80).

**Conclusion:** This study found that YKL-40 was associated with carotid plaque instability determined by ultrasound. Individuals with high YKL-40 may have a higher risk of unstable carotid plaque.

## Introduction

Stroke has already caused a heavy burden worldwide because of its high incidence, high disability rate, and high mortality, especially in China (1, 2). The rupture of a carotid atherosclerotic plaque could lead to ischemic stroke (3). YKL-40, a new inflammatory factor, is involved in the pathogenesis of atherosclerotic plaques (4). It plays a vital role in the process of matrix remodeling, cell proliferation and differentiation, new blood vessel formation, anti-apoptosis, and promotion of tissue fibrosis. Several studies have reported that high YKL-40 is associated with an increased risk of ischemic stroke, but not myocardial infarction (4–8), suggesting that YKL-40 might be a promising biomarker of plaque instability (5, 9, 10).

This study aimed to investigate the association between YKL-40 and carotid atherosclerotic plaque instability in a community-based population.

## Materials and Methods

### Study Design and Population

We enrolled 1,132 participants with carotid plaques from a community-based, cross-sectional study in Beijing from February 2014 to May 2016. The inclusion criteria for the participants were: (1) age between 24 and 75 years; (2) carotid atherosclerotic plaques detected using carotid artery color-ultrasonography; and (3) signed written informed consent was obtained. Individuals with (1) severe inflammatory diseases such as acute and chronic infections, rheumatoid arthritis, osteoarthritis, and liver cirrhosis; (2) a history of stenting, percutaneous coronary stenting, and coronary artery bypass grafting; (3) severe clinical conditions including a recent trauma, surgery, severe heart failure, hepatic and renal insufficiency, autoimmune diseases, hematologic diseases, cerebrovascular diseases, and peripheral vascular diseases; and (4) any known malignant tumors were excluded from the study.

This study was approved by the Beijing Tiantan Hospital Research Ethics Committee. All participants provided signed written informed consent to participate in this study.

### Data Collection

All participants were interviewed face-to-face with a structured questionnaire by trained interviewers. The questionnaire included questions on demographics (sex, age, body mass index, cigarette smoking, and alcohol consumption), medical history (diabetes mellitus, hypertension, dyslipidemia, coronary heart disease, and atrial fibrillation), and medications taken in the last 12 months. Height, weight, systolic blood pressure, and diastolic blood pressure were measured using standard operating procedures.

Fasting venous blood samples were drawn for routine blood examinations, measurement of lipid, fasting blood glucose, hypersensitive C-reactive protein (hsCRP), and homocysteine.

### Assessment of Carotid Plaque

Ultrasound examinations were performed by trained and certified sonographers using standard equipment (iU22 xMatrix, Philips). Bilateral carotid arteries were scanned, focusing on the near and far walls. The scanning range was 15 mm before and 10 mm after the bifurcation of the common carotid artery. Carotid plaque was defined as a thickness ≥ 1.5 mm measured from the media-adventitia interface to the intima-lumen interface or a focal structure that encroaches into the arterial lumen for at least 0.5 mm or 50% of the surrounding intima-media thickness (IMT) value (11). Based on the morphology and echogenicity of the plaques detected by ultrasound, they were categorized as (1) hypoechoic lipid soft spots; (2) moderately echogenic fibrous flat plaques abundant in collagen tissue; (3) strong acoustic echo-like calcified hard plaques; or (4) ulcerative mixed plaques with different echoes. In this study, plaques with hypoechoic or heterogeneous echoes were defined as unstable (12).

### Measurement of YKL-40

The serum YKL-40 levels were measured by an enzyme-linked immunosorbent assay (R&D Systems, China). We used the mean value of duplicate measurements. The detection limit was 20 ng/mL, while the intra-assay and inter-assay coefficients of variation were both <6%.

### Statistical Analysis

Continuous variables with normal distribution were expressed as means ± standard deviations (SDs). Non-normal variables were presented as median (inter-quartile range). Categorical variables were expressed as frequency and percentage. The student's *t*-test and Wilcoxon test were used to evaluate the difference between groups of continuous variables. Categorical variables were compared using the χ^2^ tests (the chi-squared tests). We performed univariate and multivariate logistic regression analysis to evaluate the association between YKL-40 and carotid artery plaque instability. While model 1 was unadjusted, model 2 was adjusted for age and sex, and model 3 was adjusted for the variables in model 2 plus BMI, medical history of hypertension, diabetes, coronary heart disease, dyslipidemia, smoking, and alcohol consumption. Model 4 was adjusted for the variables in model 3 plus fasting blood glucose, triglyceride, low-density lipoprotein, high-density lipoprotein, homocysteine, and high-sensitivity C-reactive protein. In Model 5, plaque thickness was added to Model 4. The significance level was defined as *P* ≤ 0.05. All statistical analyses were performed using SAS 9.4 software (SAS Institute Inc, Cary, North Carolina).

## Results

A total of 1,132 participants were recruited in this study. The mean age of the participants was 58 (52–64) years, and 560 (49.5%) of them were men. Unstable plaques were detected in 855 (75.53%) participants. Table 1 summarizes the characteristics of the participants. Compared to participants with stable plaques, those with unstable plaques were more likely to be men, and smokers. The two groups showed significant differences in plaque thickness, diabetes mellitus, dyslipidemia, white blood cell count, and levels of fasting glucose, high-density lipoprotein cholesterol and high-sensitivity C-reactive protein (Table 1). YKL-40 levels were stratified into four quartiles: quartile 1: <25.47 ng/mL, quartile 2: 25.47–39.53 ng/mL, quartile 3: 39.53–70.55 ng/mL, quartile 4: ≥70.55 ng/mL. Participants with unstable plaques had a higher median concentration of YKL-40 (40.23 ng/mL) compared to those with stable plaques (31.44 ng/mL) (Table 2).

**Table 1:** Comparison of clinical data between stable and unstable plaque groups.

**Variable**	**Total (%)**	**Stable plaque group**	**Unstable plaque group**	***p*-value**
	**(*n* = 1132)**	**(*n* = 277)**	**(*n* = 855)**	
Age (years)	58.0 (52.0–64.0)	59.0 (52.0–67.0)	58.0 (52.0–64.0)	0.24
Male, *n* (%)	560 (49.5)	108 (39.0)	452 (52.9)	<0.001
BMI (kg/m^2^)	26.1 (23.8–28.4)	25.1 (23.0–27.5)	26.4 (24.1–28.6)	<0.001
<25	417 (36.8)	134 (48.4)	283 (33.1)	<0.001
25–30	546 (48.3)	116 (41.9)	430 (50.4)	
≥30	168 (14.9)	27 (9.7)	141 (16.5)	
Smoking, *n* (%)				<0.001
Yes	804 (71.0)	51 (18.4)	277 (32.4)	
No	328 (29.0)	226 (81.6)	578 (67.6)	
Alcohol consumption, *n* (%)				0.99
Yes	413 (36.5)	101 (36.5)	312 (36.5)	
No	719 (63.5)	176 (63.5)	543 (63.5)	
Medical history, *n* (%)				
Diabetes mellitus	167 (14.8)	29 (10.5)	138 (16.1)	0.02
Hypertension	469 (41.4)	117 (42.2)	352 (41.2)	0.75
Dyslipidemia	209 (18.5)	84 (30.3)	125 (14.6)	<0.001
Coronary heart disease	67 (5.9)	15 (5.4)	52 (6.1)	0.68
Atrial fibrillation	12 (1.1)	5 (1.8)	7 (0.8)	0.16
Laboratory examination				
White blood cell count (×10^12^/L)	6.04 (5.17–7.20)	5.60 (4.86–6.85)	6.20 (5.24–7.30)	<0.001
Fasting glucose (mmol/L)	5.80 (5.20–6.64)	5.25 (4.60–6.00)	5.96 (5.40–6.86)	<0.001
Triglycerides (mmol/L)	1.39 (1.00–1.99)	1.44 (1.03–2.01)	1.38 (0.98–1.96)	0.15
Total cholesterol (mmol/L)	5.23 (4.55–5.90)	5.21 (4.54–5.75)	5.24 (4.58–5.96)	0.06
High-density lipoprotein cholesterol (mmol/L)	1.44 (1.23–1.70)	1.49 (1.28–1.74)	1.43 (1.22–1.68)	0.02
Low-density lipoprotein cholesterol (mmol/L)	3.20 (2.62–3.72)	3.22 (2.67–3.60)	3.18 (2.62–3.75)	0.80
Homocysteine (umol/L)	14.7 (12.3–18.4)	14.9 (12.5–18.2)	14.7 (12.2–18.5)	0.68
High-sensitivity C-reactive protein (mg/ml)	1.7 (1.0–3.2)	1.4 (0.8–3.0)	1.8 (1.1–3.2)	<0.001
Plaque thickness (mm)	2.20 (1.80–2.70)	1.99 (1.60–2.40)	2.20 (1.80–2.70)	<0.001

*BMI indicates body mass index, BMI (kg/m^2^) = weight/height^2^*.

**Table 2:** Comparison of YKL-40 levels between stable and unstable plaque groups.

**Variable**	**Total (%)**	**Stable plaque group**	**Unstable plaque group**	***p*-value**
	**(*n* = 1,132)**	**(*n* = 277)**	**(*n* = 855)**	
YKL-40 (ng/mL)	37.61 (24.87–62.52)	31.44 (18.96–52.72)	40.23 (26.53–65.93)	<0.001
<25.47	297 (26.24)	100 (36.10)	197 (23.04)	<0.001
25.47–39.53	302 (26.68)	81 (29.24)	221 (25.85)	
39.53–70.55	291 (25.70)	49 (17.69)	242 (28.30)	
≥70.55	242 (21.38)	47 (16.97)	195 (22.81)	

The univariate logistic analysis found male sex, history of diabetes mellitus, dyslipidemia, BMI, and plaque thickness associated with unstable plaques. Furthermore, YKL-40 levels (quartile 3: OR 2.51 95% CI 1.70–3.70 and quartile 4: OR 2.11 95% CI 1.41–3.14) were also associated with unstable carotid plaques (Table 3, Model 1).

**Table 3:** Logistic regression analysis of the association between YKL-40 and carotid atherosclerotic plaque instability.

	**Model 1**		**Model 2**		**Model 3**		**Model 4**		**Model 5**	
	**OR (95% CI)**	***p***	**OR (95% CI)**	***p***	**OR (95% CI)**	***p***	**OR (95% CI)**	***p***	**OR (95% CI)**	***p***
Q1	Reference	–	Reference	–	Reference	–	Reference	–	Reference	–
Q2	1.39 (0.98–1.97)	0.07	1.38 (0.97–1.96)	0.08	1.43 (0.99–2.07)	0.06	1.32 (0.90–1.93)	0.16	1.15 (0.74–1.79)	0.54
Q3	2.51 (1.70–3.70)	<0.001	2.55 (1.71–3.81)	<0.001	2.71 (1.78–4.11)	<0.001	2.53 (1.65–3.89)	<0.001	2.10 (1.29–3.40)	0.003
Q4	2.11 (1.41–3.14)	<0.001	2.18 (1.45–3.29)	<0.001	2.14 (1.39–3.28)	<0.001	1.98 (1.27–3.11)	<0.01	1.70 (1.04–2.80)	0.036

*Model 1 is unadjusted*.

*Model 2 is adjusted for age and gender*.

*Model 3 is adjusted for model 2 and BMI, medical history of hypertension, diabetes, coronary heart disease, dyslipidemia, smoking, and alcohol consumption*.

*Model 4 is adjusted for model 3 and fasting blood glucose, triglyceride, low-density lipoprotein, high-density lipoprotein, homocysteine and high-sensitivity C-reactive protein*.

*Model 5 adjusted for model 4 and plaque thickness*.

The multivariate logistic analysis showed an age- and sex-adjusted association between YKL-40 and carotid plaque instability in Model 2 (quartile 3: OR 2.55; 95% CI 1.71–3.81; quartile 4: OR 2.18 95% CI 1.45–3.29). After adjustment for age, sex, BMI, medical history of hypertension, diabetes, coronary heart disease, dyslipidemia, smoking, and alcohol consumption, YKL-40 level remained significantly associated with carotid plaque instability (quartile 3: OR 2.71, 95% CI 1.78–4.11; quartile 4: OR 2.14, 95% CI 1.39–3.28) in Model 3. The association remained statistically significant after adjusting for all variables in Model 4 (quartile 3: OR 2.53, 95% CI 1.65–3.89; quartile 4: OR 1.97, 95% CI 1.27–3.11), and Model 5 (quartile 3: OR 2.10, 95% CI 1.29–3.40; quartile 4: OR 1.70, 95% CI 1.04–2.80).

## Discussion

This study investigated the association between YKL-40 level and carotid plaque instability in a community-based population. We found that high-levels of YKL-40 were associated with carotid plaque instability.

Recently, several studies have explored the relationship between YKL-40 and atherosclerotic plaque instability. Michelsen et al. found that serum YKL-40 was significantly elevated in patients with carotid atherosclerosis, especially in symptomatic patients, suggesting it may be a marker of plaque instability by causing macrophage activation and matrix degradation (9). However, this study had a small size and lack prospective data. Wu et al. evaluated YKL-40 in 168 patients with carotid atherosclerosis and found thicker carotid intima-media and more unstable plaques in patients with high serum levels of YKL-40. However, these findings were limited to patients with Helicobacter pylori-positive cytotoxic-associated gene A (CagA), and therefore, the association might have been related to Helicobacter pylori infection, which needs to be further verified (13).

It is well-known that the rupture of an unstable carotid atherosclerotic plaque is one of the leading causes of ischemic stroke (14, 15). Some studies have been accessed the association between YKL-40 and ischemic stroke. Kjaergaard et al. detected plasma YKL-40 in 8,899 general participants and followed them up for 18 years. They found that increase in YKL-40 led to an increased risk for ischemic stroke, but not for atherosclerosis myocardial infarction. The differences in the mechanisms of ischemic stroke and myocardial infarction indicated that YKL-40 might play a crucial role in thromboembolism rather than affecting the formation of local thrombus and atherosclerosis, suggestive of its association with plaque instability (4, 8). Rathcke et al. in a 15-years follow-up study of 2,656 Danes reported similar findings. They found that high levels of YKL-40 were associated with increased ischemic stroke-related mortality but were inversely related to the risk of ischemic heart disease (7). These studies elucidated that YKL-40 plays different roles in thromboembolism and local thrombosis by macrophage activation and matrix degradation within the atherosclerotic lesion. Recently, Hjalmarsson et al. found that patients with National Institutes of Health Stroke Scale (NIHSS) ≥ 5 had significantly higher levels of YKL-40 compared to those with a score <5, illustrating the significant association of YKL-40 with stroke severity. YKL-40, therefore, might be an important biomarker of carotid plaque instability and a warning sign for the pathogenesis and prognosis of acute ischemic stroke (16), which is consistent with our results.

YKL-40, a biomarker of inflammation, may be involved in the pathological progression of atherosclerosis (13, 17–19). Inflammation plays an important role in atherosclerosis and atherothrombotic events. An *in vivo* study by Rathcke and Vestergaard further confirmed the expression of YKL-40 protein in the smooth muscle cells in human atherosclerotic plaques (20). YKL-40 regulated angiogenesis and reorganization of the extracellular matrix by controlling vascularized smooth muscle cells and vascular endothelial cells. An increase in YKL-40 levels activate endothelial cells to express vascular adhesion molecule-1 (VCAM-1) and intercellular adhesion molecule-1 (ICAM-1), further injuring the vascular endothelial cells, and promoting the development of atherosclerosis (20–23). Boot et al. and Fach et al. showed an increase in YKL-40 expression in the macrophages in atherosclerotic plaques (24, 25). YKL-40 potentially is involved in macrophage activation and matrix degradation within the atherosclerotic lesion, supporting its role in thromboembolisms (7).

Our study has several limitations. First is its cross-sectional design, with no follow-up information on stroke events. Therefore, only an association and not a causal relationship between YKL-40 levels and carotid atherosclerotic plaque instability could be investigated. Second, as all the study participants were recruited from a community in Beijing, there may have been a selection bias. These findings, therefore, need to be verified in a more representative community-based population study. Third, the sample size was not large enough. Large-scale, more representative, and prospective studies are needed to further investigate the role of YKL-40 in carotid atherosclerotic plaque formation and obtain higher-level evidence. Fourth, we did not collect data on medication, which could be a potential confounder because stain and antiplatelet therapy might affect the plaques' features. Fifth, in our study, the prevalence of unstable carotid plaque was 75.53%, which is a little higher than in a previous study (26). This discrepancy could be due to differences in sex distribution and the definition of plaque instability. Finally, in this study, plaque instability was defined by ultrasound, which can be largely influenced by the operator's skills, limiting the generalizability of our results. However, to minimize the functional heterogeneity, the ultrasound operators in this study were required to have more than five years of clinical experience. We also conducted uniform standardized training for all ultrasound operators before this study.

In conclusion, this study found that YKL-40 is associated with carotid plaque instability. High levels of YKL-40 are associated with a higher risk of carotid plaque instability, suggesting that YKL-40 is potentially a marker for plaque instability.

## Data Availability Statement

The datasets used and analyzed during the current study will be made available by the corresponding author upon reasonable request.

## Ethics Statement

The studies involving human participants were reviewed and approved by the Beijing Tiantan Hospital research ethics committee. The patients/participants provided their written informed consent to participate in this study.

## Author Contributions

GL designed and conceived the study. YW and BL analyzed the data and drafted the article. YJ, RZ, and XZ contributed in the data collection and interpretation. XM, XZ, and YW critically revised the manuscript. All authors approved the final version of the article.

## Conflict of Interest

The authors declare that the research was conducted in the absence of any commercial or financial relationships that could be construed as a potential conflict of interest.
